# Patient-reported outcomes of laser hair removal for hidradenitis suppurativa: an exploratory cross-sectional survey

**DOI:** 10.1007/s10103-026-04893-6

**Published:** 2026-06-25

**Authors:** Leandro Bosch, Lynna Yang, Kelsey Shannon Flood, Julia Mhlaba Riley

**Affiliations:** Department of Dermatology, Northwestern University Feinberg School of Medicine, Chicago, USA

**Keywords:** Hidradenitis suppurativa, Laser hair removal, Patient-reported outcomes, Survey, REDCap

## Abstract

Purpose: Hidradenitis suppurativa (HS) is a chronic, debilitating skin disease often requiring multimodal therapy. Laser hair removal (LHR) is an emerging treatment option, yet patient-centered data is limited. This study aimed to assess patient perspectives on the effectiveness, safety, motivations, and barriers associated with LHR for HS. Methods: An anonymous cross-sectional online survey was administered via REDCap (July–December 2024) to adults with HS living in the United States. Respondents reported prior treatments, LHR parameters, outcomes, adverse effects, and barriers. Descriptive statistics were used. Results: Of 110 participants who completed the survey (110/132, 83%), 24 (22%) had used LHR and comprised the analytic cohort, with a median of 8 LHR sessions (IQR 6–12). Leading motivations included reducing inflammation (92%), relieving pain (75%), and seeking durable treatment (71%). Highest median improvements (score 4, IQR 4–5) were in lumps/abscesses, swelling, and flare frequency. Other symptoms, including pain, odor, and quality of life, also showed moderate improvement. Half reported benefits lasting over 12 months. While biologics were perceived as most effective (median 4.5, IQR 3.5–5), LHR received one of the highest median scores among non-biologic options (3.5, IQR 3–5). Barriers included cost, insurance limitations, and low awareness; 63% paid over $1,000, and 38% discontinued early. Common adverse effects included discomfort (71%) and transient erythema (46%). Conclusion: Most patients perceived LHR as beneficial for HS, but affordability and awareness remain barriers. Findings highlight the need for payer advocacy and additional trials defining LHR’s role in HS management.

## Introduction

 Hidradenitis suppurativa (HS) is a chronic inflammatory skin disease characterized by recurrent painful nodules, abscesses, sinus tracts, and scarring [1]. The disease severely impacts quality of life, causing physical discomfort, psychological distress, and social stigma [2, 3]. Current treatments, including topical therapies, antibiotics, hormonal interventions, and biologics, often yield inconsistent results and significant side effects. Surgical options are helpful adjunctive treatments but pose risk of recurrence, complicated and intensive healing, and substantial morbidity [4, 5].

Laser hair removal (LHR) targets hair follicles to reduce follicular occlusion, a primary pathogenic mechanism in HS [3, 6–9]. Emerging evidence supports LHR as an effective and safe therapeutic alternative, yet utilization remains low, largely due to limited patient and provider awareness, challenges with insurance coverage, and a lack of patient‑reported data [4, 5, 10–12]. This exploratory survey aimed to assess patient perspectives regarding the effectiveness, satisfaction, motivations, and barriers associated with LHR treatment for HS.

## Methods

### Study design and participants

An anonymous cross-sectional online survey was administered via REDCap between July and December 2024. Participants were eligible if they were English-speaking adults (≥ 18 years) residing in the United States with a diagnosis of HS. Recruitment was conducted through ResearchMatch and online HS support communities (including social media platforms).

### Survey instrument

The questionnaire was reviewed and refined by all authors for content relevance and clarity, and pretested for usability and technical functionality among co-authors before distribution. It collected demographic and clinical characteristics, including HS severity, Hurley stage, prior and current treatments, and LHR experiences. For respondents with prior LHR use, the survey assessed motivations, treatment characteristics, perceived outcomes, adverse effects, perceived effectiveness, and barriers. Respondents without prior LHR use completed a separate set of questions on nonuse. The survey was administered in REDCap using branching logic to display relevant items based on prior LHR experience.

### Analysis

Data from respondents who had undergone LHR were analyzed descriptively using medians, interquartile ranges (IQRs), means, and proportions. Exploratory subgroup analyses by age and Hurley stage were conducted; given small sample sizes, these are hypothesis-generating only and no statistical inference was performed. Only fully complete surveys with all forms marked as complete were included in analysis (*n* = 110). Partially complete surveys (*n* = 22) were excluded.

## Results

### Participant characteristics

Of 132 individuals who consented to participate, 110 completed all survey forms (83% completion rate). Twenty‑four (22% [24/110]) reported LHR experience and comprised the analytic cohort. Respondents were predominantly self-identified female (92% [22/24]), White (79% [19/24]), non-Hispanic (83% [20/24]), and most reported moderate HS severity (54% [13/24]; Table 1). 

**Table 1:** Demographic and clinical characteristics of 24 individuals with hidradenitis suppurativa (HS) who use laser hair removal

Characteristic	Value
Age (years)	Mean 36.1 (SD 7.2)
	Median 38.5 [IQR 31.0–43.7]
	Range 21–51
Gender Identity	Female: 22 (92%)
	Nonbinary: 2 (8%)
Race	White: 19 (79%)
	Black or African American: 4 (17%)
	Middle Eastern: 1 (4%)
	Preferred not to specify: 1 (4%)
Ethnicity	Hispanic: 4 (17%)
	Non-Hispanic: 20 (83%)
U.S. Region of Residence	South: 8 (33%)
	West: 7 (29%)
	Midwest: 4 (16%)
	Northeast: 3 (12%)
	Noncontiguous US: 2 (8%)
Marital Status	Married/Engaged: 11 (46%)
	Single: 7 (29%)
	Other: 6 (25%)
Income	$100–199k: 7 (29%)
	$200k+: 6 (25%)
	Other: 11 (46%)
Insurance	Employer-sponsored: 18 (75%)
	Individual: 3 (12%)
	Government: 3 (12%)
Fitzpatrick skin phototype	Type I–II: 10 (41%)
	Type III: 8 (33%)
	Type IV–V: 6 (25.0%)
HS Onset Age	Mean 18.5 (SD 7.1), Range 7–36
	Median 15.5 years (IQR 13.0–22.3)
HS Diagnosis Age	Mean 27.8 (SD 9.5), Range 13–47
	Median 26.0 years (IQR 20.8–35.5)
HS Severity	Moderate: 13 (54%)
	Mild: 9 (38%)
	Severe: 2 (8%)
HS Stage	Stage I: 6 (25%)
	Stage II: 16 (66%)
	Stage III: 2 (8%)
Flare Frequency	Several/month: 9 (38%)
	Several/year: 9 (38%)
	Daily: 4 (17%)
	Weekly: 2 (8%)
Flare Duration	≥ 1 week: 13 (54%)
	1–3 days: 6 (25%)
	3–7 days: 5 (21%)

HS severity and Hurley stage were collected as separate self-reported items; no standardized clinical scoring tool was applied

Demographic and clinical characteristics of participants with hidradenitis suppurativa (HS) who underwent laser hair removal (LHR) (N = 24). Data are presented as n (%) unless otherwise stated. IQR, interquartile range. All data is self-reported

Abbreviations: *HS* hidradenitis suppurativa, *LHR* laser hair removal, *SD* standard deviation, *IQR* interquartile range

### Perceived outcomes

**Fig. 1:**
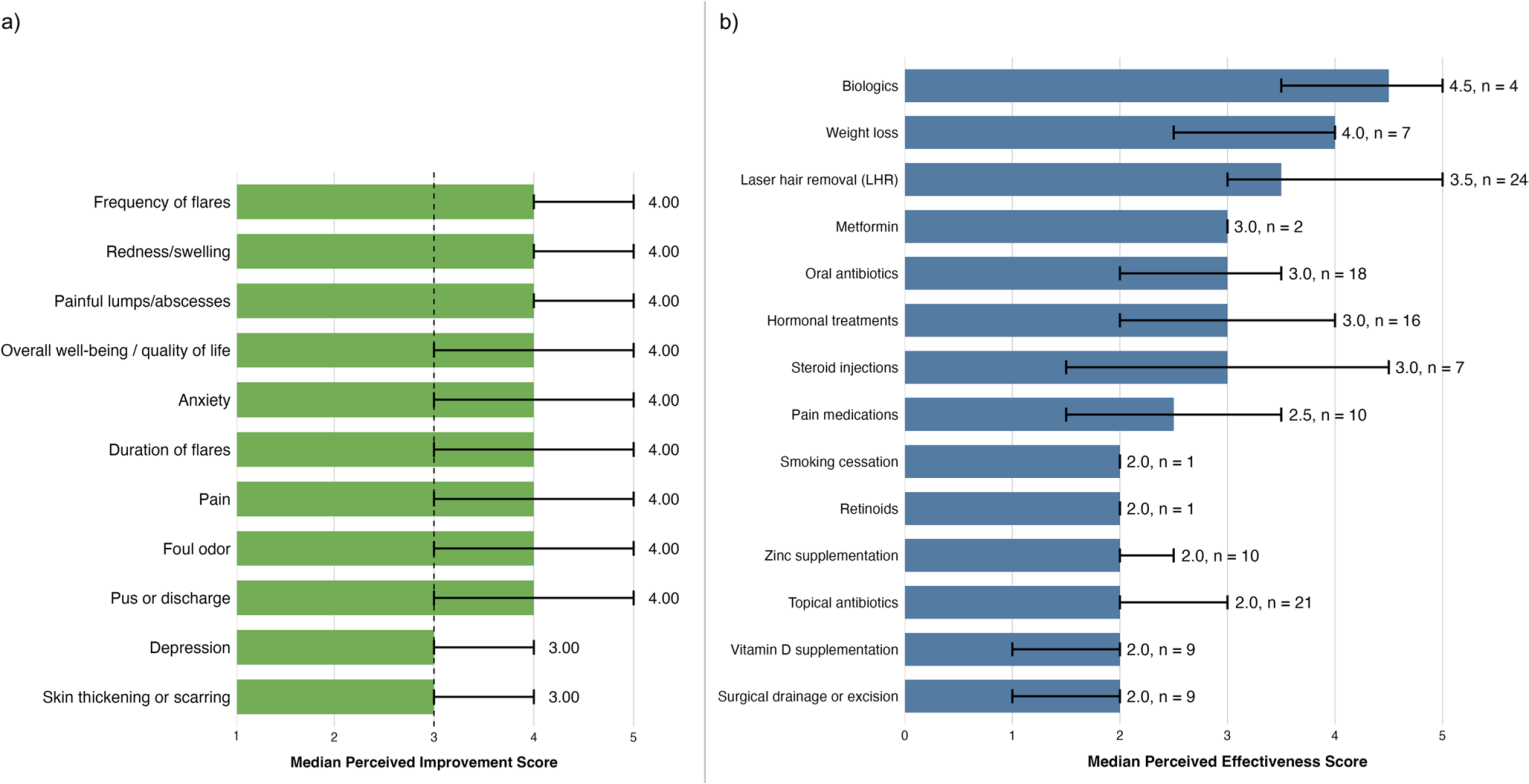
Patient-Reported Effectiveness of Laser Hair Removal (LHR) for Hidradenitis Suppurativa (HS) (**a**) Median perceived improvement scores (with interquartile ranges [IQRs]) by symptom domain following LHR (n = 24). Scores range from 1 (“significantly worse”) to 5 (“significantly better”), with 3 indicating “no change” (**b**) Median perceived effectiveness ratings (with interquartile ranges [IQRs]) of LHR compared to other HS treatments. Participants rated each modality on a 0–5 scale, where 0 = “not effective” and 5 = “highly effective.” The number of respondents (n) varies by treatment and is indicated for each bar in parentheses

Respondents underwent a median of 8 LHR sessions (IQR 6–12), with improvement reported after a median of 3 sessions (IQR 1.75–6.25; Table 2). Participants reported the greatest improvement in painful lumps, redness/swelling, and flare frequency, each with a median improvement score of 4 (IQR 4–5) on a 1–5 scale. Pus/discharge, foul odor, pain, flare duration, anxiety, and overall well-being/quality of life also improved, each with a median score of 4 (IQR 3–5) (Fig. 1a). Half (12/24) reported symptom relief lasting more than 12 months. Laser hair removal had a median participant-reported effectiveness rating of 3.5 (IQR 3.0–5.0; *n* = 24), following biologics (median 4.5, IQR 3.5–5.0; *n* = 4) and weight loss (median 4.0, IQR 2.5–4.0; *n* = 7) (Fig. 1b).

**Table 2:** Patient’s experience with laser hair removal (LHR) for HS (N = 24)

Survey domain	Category	Value
Total number of LHR Sessions	Median (IQR)	8 (6–12)
Sessions Before Improvement	Median (IQR)	3 (1.75–6.25)
Duration of Improvement	< 1 month	17% (*n* = 4)
	1–3 months	17% (*n* = 4)
	3–6 months	8% (*n* = 2)
	6–12 months	8% (*n* = 2)
	> 12 months	50% (*n* = 12)
Additional Outcomes Reported	Hair regrowth	21% (*n* = 5)
Early Discontinuation	Stopped early	38% (*n* = 9)
	Continued as recommended	63% (*n* = 15)
Total Out of Pocket Cost of LHR	Less than $100	4% (*n* = 1)
	$100–499	8% (*n* = 2)
	$500–999	25% (*n* = 6)
	$1000 or more	63% (*n* = 15)
Awareness of Insurance Coverage	Yes	50% (*n* = 12)
	No	50% (*n* = 12)
Likelihood to Recommend LHR	Very likely	58% (*n* = 14)
	Likely	21% (*n* = 5)
	Neutral	8% (*n* = 2)
	Unlikely	0% (*n* = 0)
	Very unlikely	13% (*n* = 3)

All data is self-reported

Abbreviations: *HS* hidradenitis suppurativa, *LHR* laser hair removal, *SD* standard deviation

In exploratory subgroup analyses, participant-reported LHR effectiveness ratings were higher among those with lower Hurley stage disease than among those with Hurley stage III disease, although the numbers were small and uneven, precluding meaningful statistical inference (Table 3). Because no statistical comparisons were performed and IQRs across treatment groups were wide and overlapping, these rankings should be interpreted as descriptive only and do not imply meaningful differences in effectiveness between modalities.

**Table 3:** Effectiveness ratings of HS treatments stratified by age group (A) and Hurley stage (B)

A) Age-stratified ratings			
Treatment	≤ 35 (*n* = 10)	36–40 (*n* = 8)	≥ 41 (*n* = 6)
Biologics	4.5 (4–5), *n* = 2	5 (5–5), *n* = 1	3 (3–3), *n* = 1
Laser Hair Removal	4 (3–5), *n* = 10	4 (3–5), *n* = 8	3 (1–4), *n* = 6
Hormonal Treatments	4 (2–4), *n* = 7	2.5 (1–3), *n* = 4	3 (3–4), *n* = 5
Steroid Injections	4 (2–5), *n* = 4	1 (1–1), *n* = 1	3 (2–4), *n* = 2
Metformin	4 (4–4), *n* = 1	2 (2–2), *n* = 1	—
Oral Antibiotics	2.5 (1.5–3), *n* = 8	3 (2–4.5), *n* = 4	3.5 (3–4), *n* = 6
Topical Antibiotics	2 (1–3), *n* = 8	2 (2–3), *n* = 7	2 (2–4), *n* = 6
Pain Medications	2 (1–3), *n* = 4	3 (1–4), *n* = 3	3 (1–5), *n* = 3
Weight Loss	2 (2–4), *n* = 3	4 (3–4), *n* = 3	4 (4–4), *n* = 1
Zinc Supplementation	2 (0–2), *n* = 3	2 (1–3), *n* = 3	2.5 (2–3.5), *n* = 4
Surgical Drainage or Excision	1.5 (0–3), *n* = 2	1.5 (0.5–2.5), *n* = 4	2 (1–2), *n* = 3
Vitamin D supplementation	1 (0–2), *n* = 2	2 (1–3), *n* = 2	2 (2–2), *n* = 5
Retinoids	—	2 (2–2), *n* = 1	—
Smoking Cessation	—	—	2 (2–2), *n* = 1
B) Hurley stage–stratified ratings			
Treatment	Hurley Stage I (*n* = 6)	Hurley Stage II (*n* = 16)	Hurley Stage III (*n* = 2)
Laser Hair Removal	5 (4–5), *n* = 6	3 (3–4.5), *n* = 16	2 (1–3), *n* = 2
Steroid Injections	5 (5–5), *n* = 1	3.5 (2–4.5), *n* = 4	1.5 (1–2), *n* = 2
Hormonal Treatments	3 (2.5–3.5), *n* = 4	3 (2–4), *n* = 10	3.5 (3–4), *n* = 2
Weight Loss	3 (2–4), *n* = 3	4 (3–4), *n* = 4	—
Metformin	3 (2–4), *n* = 2	—	—
Pain Medications	3 (3–3), *n* = 1	2 (1–3), *n* = 7	4.5 (4–5), *n* = 2
Topical Antibiotics	2 (2–2), *n* = 5	2 (1–3), *n* = 14	2 (2–2), *n* = 2
Oral Antibiotics	2 (1–3), *n* = 4	3 (2.5–4), *n* = 12	3 (3–3), *n* = 2
Zinc Supplementation	1.5 (1–2), *n* = 2	2.5 (2–3), *n* = 6	2 (2–2), *n* = 2
Vitamin D Supplementation	0.5 (0–1), *n* = 2	2 (2–3), *n* = 5	2 (2–2), *n* = 2
Surgical Drainage or Excision	0 (0–0), *n* = 1	1.5 (1–2), *n* = 6	2.5 (2–3), *n* = 2
Biologics	—	5 (4–5), *n* = 3	3 (3–3), *n* = 1
Retinoids	—	2 (2–2), *n* = 1	—
Smoking Cessation	—	—	2 (2–2), *n* = 1

Values are presented as median (interquartile range [IQR]) of effectiveness ratings on a 0–5 scale, where 0 = not effective and 5 = highly effective. The number of respondents (n) contributing to each estimate is shown

Abbreviations: *HS* hidradenitis suppurativa, *LHR* laser hair removal

### Adverse effects

**Fig. 2:**
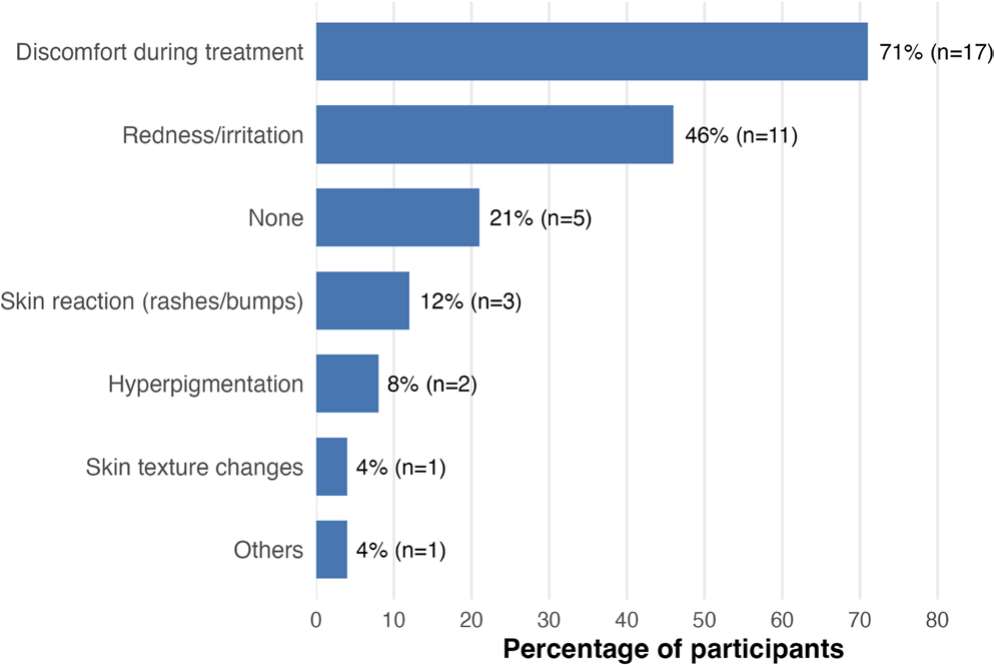
Patient-reported side effects of laser hair removal (LHR) for hidradenitis suppurativa (HS) (*n* = 24). Bars show the percentage of participants endorsing each adverse effect

Treatment‑related discomfort was most common (71% [17/24]), followed by redness/irritation (46% [11/24]) and skin reactions (13% [3/24]). Hyperpigmentation (8% [2/24]) and textural changes (4% [1/24]) were rare. One in five participants reported no side effects (Fig. 2).

### Motivations for pursuing LHR

**Fig. 3:**
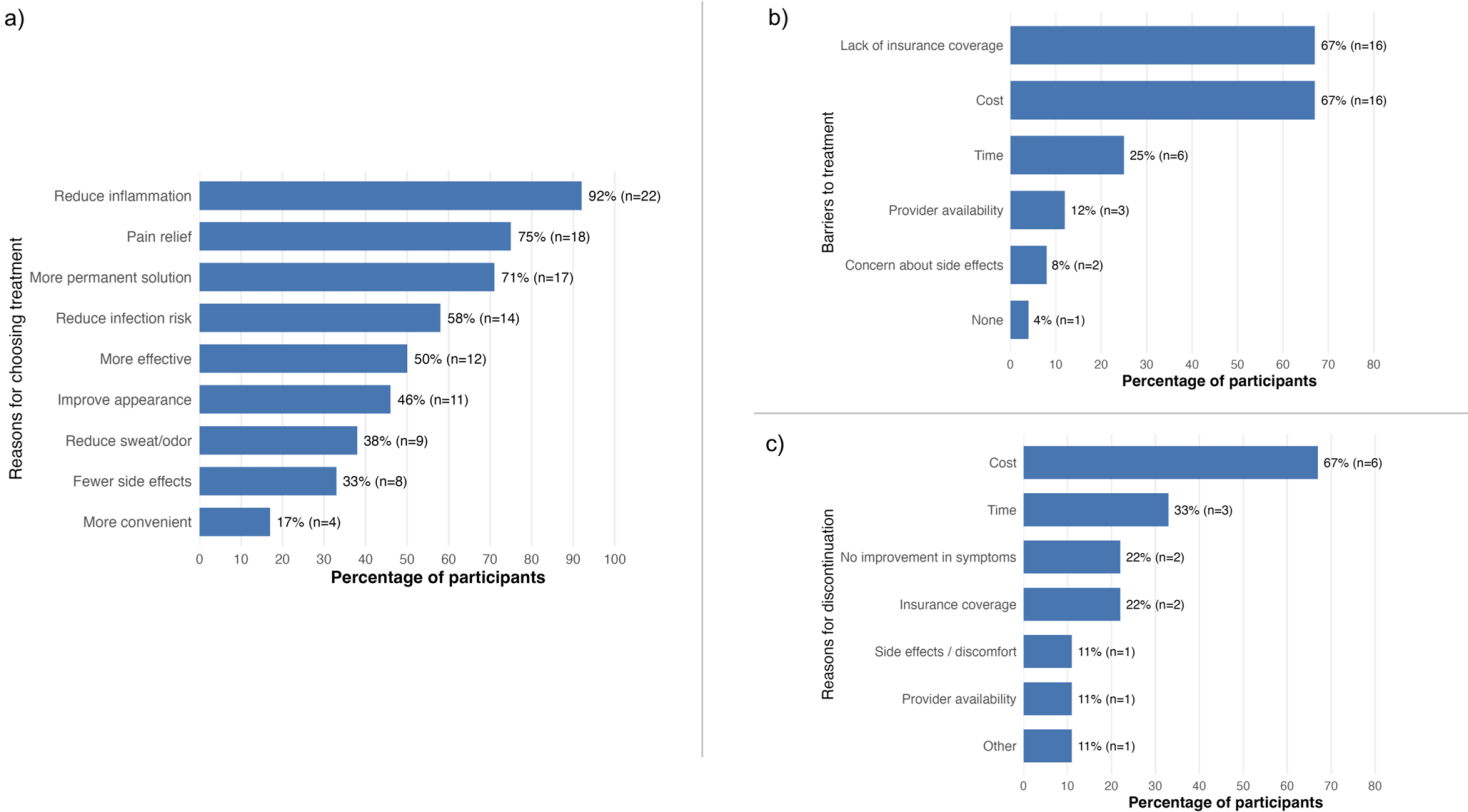
Patient-Reported Motivations, Barriers, and Discontinuation Reasons Related to Laser Hair Removal (LHR) for Hidradenitis Suppurativa (HS) (**a**) Factors influencing participants’ decision to pursue LHR treatment (n = 24) (**b**) Barriers to accessing or continuing LHR, as reported by participants (n = 24) (**c**) Reasons for early discontinuation of LHR among participants who did not complete the full course as recommended (n = 9)

Participants most commonly pursued LHR to reduce inflammation (92% [22/24]), relieve pain (75% [18/24]), and achieve a more durable solution (71% [17/24]). Many (67% [16/24]) also reported turning to LHR after feeling they had already tried ‘everything else’, whereas provider recommendation and social media were less common motivators (25% each [6/24]; Fig. 3a).

### Barriers, cost, and early discontinuation

Financial burden was the dominant barrier to LHR. Two-thirds of participants (67% [16/24]) cited cost or lack of insurance coverage, and 63% reported out-of-pocket expenses exceeding US$1,000. More than one-third (38% [9/24]) discontinued treatment early, most commonly because of cost (67% [6/9]). Half of respondents were unaware that LHR may be covered by insurance for HS. Discomfort during treatment (50% [12/24]) and time constraints (25% [6/24]) were also reported barriers (Fig. 3b, c; Table 2).

## Discussion

This exploratory survey provides patient-reported data suggesting that LHR may be a meaningful adjunctive treatment for HS. LHR is thought to act through selective photothermolysis of the follicular unit implicated in HS pathogenesis. Prior controlled studies and North American guideline recommendations also support a therapeutic role for LHR in HS [1–3, 6]. Against this background, our findings contribute early patient-reported, real-world data suggesting that LHR may help improve multiple HS-related symptoms and quality-of-life domains, while also highlighting the limited accessibility of this modality in routine HS care.

Participants assigned LHR a favorable effectiveness rating (median 3.5, IQR 3.0–5.0). These findings suggest that patients view LHR as a potentially useful adjunctive option within the HS treatment landscape. This may be particularly relevant given the access barriers associated with biologics [11] and concerns about antimicrobial resistance and the cumulative burden of long-term systemic therapies [13, 14]. Wider adoption may potentially reduce pain, reliance on opioid analgesics, and other adjuncts that drive morbidity in HS care.

The greater perceived benefit reported among participants with lower Hurley stages (Table 3) is consistent with prior mechanistic and clinical observations that LHR may be more beneficial earlier in the disease course, before extensive scarring and sinus-tract formation occur [4, 10, 15]. Because LHR targets the follicular unit implicated in early HS pathogenesis, it may be particularly relevant in less advanced disease. This interpretation remains hypothesis-generating, and prospective longitudinal studies are needed to determine whether earlier use of LHR is associated with improved long-term outcomes or altered disease progression.

Only a minority of surveyed patients reported prior LHR use, suggesting that this modality may remain underutilized in HS care. Despite perceived benefits, financial barriers were substantial: two‑thirds of respondents cited lack of insurance coverage or cost, and many discontinued treatment early, most commonly because of cost. Half were unaware that insurance coverage might be possible, highlighting a potentially modifiable gap in clinician counselling and patient education [16]. As evidence for the benefits of LHR in HS continues to develop, professional societies and payers should continue to revisit coverage policies. Improving access may be particularly important for patients with limited financial resources, who often bear a disproportionate burden of HS.

### Limitations and recommendations

The modest number of respondents with prior LHR use limits generalizability, although the small number of LHR users may also reflect the limited real-world uptake of this modality. The absence of male respondents further narrows the applicability of these findings. Additional limitations include the study’s reliance on patient-reported outcomes. Because medical records were not reviewed, consistent with the survey-based design, diagnoses and clinical characteristics could not be independently verified. Recruitment through HS-focused communities may also have introduced selection bias by preferentially capturing more engaged patients, and retrospective self-report introduces the potential for recall bias. Because temporal data on other HS treatments were not collected, perceived benefits may have been influenced by concomitant therapies. Incomplete surveys were excluded, and reasons for noncompletion were not formally assessed; however, potential explanations include survey fatigue, time constraints, or technical difficulties, as some participants completed only the initial pages before discontinuing. The absence of validated objective outcome measures, such as IHS4 or HiSCR, reduces comparability with clinical trial data. In addition, data on laser type, treatment parameters, and anatomic sites treated were not collected, limiting comparison with prior clinical studies. Subgroup analyses, including those stratified by age and Hurley stage, should be interpreted cautiously because of the small numbers in several strata. Accordingly, these findings should be interpreted as exploratory and hypothesis-generating rather than as evidence of objective efficacy. Future prospective studies incorporating objective disease metrics and larger, more diverse cohorts are needed to clarify how perceived benefit varies by disease severity and patient characteristics and to better define optimal patient selection and treatment protocols.

## Conclusion

LHR was perceived by surveyed patients as a potentially beneficial adjunctive treatment for HS, particularly for pain and inflammatory symptoms, but its use remains limited by cost and access barriers. These exploratory findings support further prospective study to better define the role of LHR in HS management. 

## Data Availability

De-identified survey data are available from the corresponding author upon reasonable request.
